# Measuring Energetics and Behaviour Using Accelerometry in Cane Toads *Bufo marinus*

**DOI:** 10.1371/journal.pone.0010170

**Published:** 2010-04-21

**Authors:** Lewis G. Halsey, Craig R. White

**Affiliations:** 1 School of Life Sciences, Roehampton University, London, United Kingdom; 2 School of Biological Sciences, The University of Queensland, St Lucia, Queensland, Australia; Universidad Europea de Madrid, Spain

## Abstract

Cane toads *Bufo marinus* were introduced to Australia as a control agent but now have a rapidly progressing invasion front and damage new habitats they enter. Predictive models that can give expansion rates as functions of energy supply and feeding ground distribution could help to maximise control efficiency but to date no study has measured rates of field energy expenditure in an amphibian. In the present study we used the accelerometry technique to generate behavioural time budgets and, through the derivation of ODBA (overall dynamic body acceleration), to obtain estimates of energetics in free ranging cane toads. This represents the first time that accelerometers have been used to not only quantify the behaviour of animals but also assign to those behaviours rates of energy expenditure. Firstly, laboratory calibrations between ODBA and metabolic rate were obtained and used to generate a common prediction equation for the subject toads (R^2^ = 0.74). Furthermore, acceleration data recorded during different behaviours was studied to ascertain threshold values for objectively defining behaviour categories. Importantly, while subsequent accelerometer field deployments were relatively short they agreed with previous studies on the proportion of time that cane toads locomote yet suggest that the metabolic rate of cane toads in the wild may sometimes be considerably higher than might be assumed based on data for other species.

## Introduction

The cane toad *Bufo marinus* is an introduced species in Australia that presently inhabits the north-east of the country and has a rapidly progressing invasion front [1], [2]. This species exists at much higher densities in Australia than in its native South America [3] and is altering the ecologies in which it is now present, for example through reducing invertebrate abundance and species richness, predating on the nests of ground-nesting birds [4], and introducing protozoan parasites [5].

Given the extent of the impact of cane toads in their current range in Australia, it is of particular interest to know how far cane toads will spread and how rapidly [6], in part to inform allocation of control and conservation efforts [2]. Energetics information can provide critical information for predicting species distribution [7]. For example, data are required on how much time and energy individuals have for dispersal, and how much energy is expended in different microclimates for inclusion in models on rates of population expansion [8]. However, little is known about the energy requirements of free-ranging cane toads and thus the magnitude of their impact on Australian ecologies in terms of the amount of biota they need to ingest to fuel these requirements.

While overall energy expenditure in free-ranging animals in the field has been measured in over 300 species [9], [10], to our knowledge such data are not available for any species of amphibian. There are two presently common methods for measuring field energy expenditure: the doubly labelled water technique and the heart rate technique. The doubly labelled water technique is not applicable for amphibians because they have high rates of water turnover and low rates of carbon dioxide production [11]. The suitability of the heart rate technique for amphibians has not yet been tested, though it has proven suitable for at least some species of fish [12].

The accelerometry technique for estimating energy expenditure involves measuring the body motion of animals having calibrated the magnitude of body motion with rate of oxygen consumption. The metric of body motion employed is termed ‘overall dynamic body acceleration’ [ODBA; 13], and is derived from recordings of acceleration in the three spatial dimensions by a data logger placed on a fixed point of the animal. Significant relationships between energy expenditure and ODBA in laboratory settings have been published for ten endotherm species [13], [14], [15], [16], [17] and the technique can also be used to quantify behavioural time budgets since different behaviours tend to produce different acceleration trace signatures [18], [19]. However, in the four years since the accelerometry technique was first described [13], it has not been applied to obtain measures of metabolic rate in the wild, and a relationship has not been produced for an ectotherm.

Having accounted for temperature, ODBA should relate well with energy expenditure in post absorptive ectotherms. Furthermore, factoring in estimates of specific dynamic action [SDA; 20], those relationships should enable valid estimates of field energetics. The present study aims to (i) undertake laboratory experiments to develop ODBA-based equations for estimating energy expenditure, and ascertain threshold values for quantifying behaviour, in cane toads, and (ii) demonstrate the applicability of the technique to large, free-ranging anurans by using the laboratory data to obtain initial measures of behavioural-energetics in instrumented individuals in the wild.

## Materials and Methods

All experimental procedures were approved by the University of Queensland NEWMA Animal Ethics Committee (SIB/075/09/ARC).

Cane toads (mean mass 66.5 g, range 3.6 to 207.2 g) were caught from the Brisbane area, Australia, at night and housed at the University of Queensland, in a glass covered plastic container (95×60×45 cm deep). The container was lined with pine bark mulch and included a water bath. Room temperature during all experiments was 24–26°C. Cockroaches *Nauphoeta cinerea* were provided as food for animals not involved in respirometry trials. Resting metabolic rate (rate of oxygen consumption; V̇o_2_) was measured for all toads caught (n = 31) using respirometry once the toads had been fasted for at least a week and thus were post absorptive (dissection at the conclusion of measurements, following the sacrifice of chilled toads by double pithing, confirmed that digestive tracts were empty). The largest animals caught, four males (mean mass ± SD: 145.0±56.8 g) and five females (120.0±11.3 g), were included in further respirometry trials and also field trials. These further respirometry trials were run in two stages to obtain metabolic rates for the toads during low level activity (e.g. small movements) and high level activity (e.g. locomotion).

### Respirometry

An open-circuit respirometry system was used to measure V̇o_2_ during rest. Air was pulled through the respirometer chamber (a jar, either 12×7 cm diameter, 0.46 L or 7.5×4.5 cm diameter, 0.12 L) using a pump (TR-SS3, Sable Systems, USA) at between approximately 250 and 500 mL min^−1^ depending upon the size of the subject toad. Flow rate was measured using a mass flow meter calibrated with a NIST-traceable bubble film flow meter (1–10–500 ml, Bubble-O-Meter, USA). No equipment was attached to the toads during these experiments and the jar was placed in a covered, polystyrene box to limit disturbance and thus encourage deep rest in the animal. The time to steady state of the system was approximately 5 min. The flow was passed through a drying column (Drierite, Fisher Scientific) and then analysed for the fractional content of oxygen and carbon dioxide using appropriate gas analysers (Oxzilla II and FC-10A, Sable Systems). The drying agent had been exhausted and recharged prior to the experiments, limiting its affinity for carbon dioxide [21]. The outputs from the gas analyser, along with a thermocouple meter (TC-1000, Sable Systems) located next to the respirometer chamber, were recorded onto PC via an analogue to digital converter (Powerlab 16/30, ADInstruments, Australia) at 10 Hz, and smoothed (with a 7 sample running mean with Bartlett-weighting) and viewed using LabChart (v. 6.1.3, ADInstruments). The respirometry system was leak-tested using nitrogen injections [22].

Rate of oxygen consumption (V̇o_2_) was determined from the rate of airflow out of the respirometer and the difference in the fractional concentration of oxygen between ambient and out-flowing air. In all cases, gas concentrations were calculated as dry at standard temperature (273 K) and pressure (101.3 kPa). Resting experiments were undertaken on the same toad two or three times and at different hours of the day, since even during daylight hours, when the experiments were run, cane toad resting metabolic rates can vary with photoperiod [23]. During these experiments the toads would alternate between periods of only cutaneous respiration and periods of both cutaneous and pulmonary respiration, each of which could last for many minutes. To accurately measure steady-state metabolic rate during rest, V̇o_2_ was calculated from periods of at least one hour and where the mean respiratory exchange ratio was between 0.7 and 0.8.

To obtain V̇o_2_ data on a range of low level activity, a toad was placed in a 7 L respirometry box (21.5×32.5×10.0 cm in height), which included a layer of mulch. The box had an outlet at one end to which tubing was attached connecting the box to the gas analyser, and an outlet to ambient air at the other. Flow rate through the chamber was between 1000 and 1300 mL min^−1^. Otherwise, the system was identical to that used to obtain resting data. The toads were left for between 30 and 60 minutes and the relatively large size of the box allowed them to undertake a range of movements including slow walking/crawling forwards and backwards, burrowing into the mulch, leaning against the side of the box while standing up on their back legs, and croaking. Where the toads were being continuously sedentary, movement was sometimes induced by passing a piece of paper over the top of the box which caused the toad to move or to duck their head.

To obtain a range of high activity V̇o_2_ data (typically walking and hopping), the toad was placed in a 5.7 L open-bottom respirometry box (14.5×27×14.5 cm in height) and the same flow arrangement as the larger box (flow rate: 1500 mL min^−1^). The bottom edges of the sides of the respirometer were encased in dense foam tubing to create an air-tight seal with the smooth, painted bench top. This respirometer also included a 12 V fan connected to a 9 V battery to gently mix the chamber air. The toads were encouraged to move from the back to the front by the presence of a researcher behind the respirometer. Upon movement of the toad the respirometer was slid forwards such that the toad was again towards the back of it, encouraging the toad to move forwards again. Movement by the toads involved walking, crawling and hopping. Data were also collected for lower activity movement by leaving the toad unattended. Nitrogen leak tests (Fedak et al. 1981) while sliding the box on the bench surface indicated maximum errors of 4% in the calculation of V̇o_2_.

For the low activity and high activity respirometry experiments, measurements of V̇o_2_ were made for each two minutes beyond the first two minutes that the toad was in the respirometer box, to allow time for the animal to reach an active physiological steady-state [16], [24]. The measurements were calculated in real time by LabChart using an instantaneous equation, i.e. which calculates values of V̇o_2_ from data obtained over periods considerably less than one minute [16]; in the present study every 10 s.

### Accelerometry

During the low and high activity experiments the toads were instrumented with an accelerometry and temperature data logger. Firstly, a section of pantyhose material (YLC™, containing nylon, elastane and cotton), with the toe end cut off and the remaining garment wrapped back on itself to create a loop, was placed around the body of the toad. A firm fit on the toad was achieved by placing the material around the toad between the front and back legs, then twisting the material on the back of the toad to make a second, upper loop, and finally pulling the upper loop down over the head of the toad to fit behind the head and anterior to the front legs. This created an x-shaped cross of material on the back of the animal under which the logger could be firmly placed without restricting the movement of the animal's legs and, since the material allows diffusion of air, without restricting cutaneous gas exchange. Further, the material remained fitted to the body of the animal regardless of whether the toad expanded its lungs or not. The logger was placed broadside against the skin with the length along the antero-posterior axis and the battery attached dorsally to the logger.

The loggers used were of the same type as those in previous studies of ODBA (e.g. Wilson et al. 2006; Halsey et al. 2008). They were set to record tri-axial acceleration (0–6 *g*) at 10 Hz with 22-bit resolution onto a 128 Mb RA memory card. This recording frequency is sufficiently high when using measures of acceleration primarily as a proxy for energy expenditure [17]. The loggers were 4.0×3.0×1.0 cm maximum lengths and had a mass of 23.2 g when a 3.6 V lithium battery was attached. From the side of the logger, which was oriented towards the caudal end of the animal, protruded a temperature probe. Temperature recorded was taken to represent body temperature of the instrumented toad during activity experiments and field deployments; ambient temperature is generally an accurate indicator of body temperature in toads [25], [26]. All toads used in the activity experiments were at least 90 g in mass at the start (mean mass: 130.3; range: 94.4 to 201.8 g), thus the logger mass was between 12 and 25% that of body mass.

Data from the accelerometer and temperature loggers were downloaded onto a PC using custom-made software. The x axis of the logger measured sway, the y axis measured surge, and the z axis measured heave [see [27],[28] for more details]. From the downloaded logger data an approximation of absolute *g* resulting from only dynamic acceleration in each of the three dimensions was extracted from each axis following removal of the static acceleration using a running mean (over a period of 1 s) as described by Wilson et al. (2006). These values were then summed to produce ODBA (see Wilson et al. 2006 for more details).

### Field deployments

A miniature single-stage radio transmitter (Sirtrack, Havelock North, New Zealand) was attached to a logger before deployment to increase the chances of recapture of the logger and toad. The total mass of the deployed package was 28 g. A total of nine deployments were made during four nights (18^th^ to 21^st^ May 2009) on eight different toads. Eight recaptures on seven individuals were made (mean mass of recovered toads at deployment: 135.9 g; range: 97.3 to 204.3 g) and data were successfully recorded in each case. Total recording time while the loggers were deployed was 21 hours. The logger, and hence data, for one animal was not recovered due to a loss of signal from the radio transmitter. Three field sites were used: The University of Queensland Alumni Garden, a cricket pitch and surrounding parkland in Brisbane, and an association football pitch and surrounding parkland, also in Brisbane. Loggers deployed in the university garden were recovered the following morning while loggers deployed on the sports pitches were constantly radio tracked and recovered later the same night.

### Data analysis

Preliminary analyses were undertaken using Excel (Microsoft Corp.) with statistical analyses conducted using JMP (v. 7, SAS Institute Inc.).

A relationship to predict resting metabolic rate from body mass was generated from a single linear regression of log(V̇o_2_) against log(body mass) for 31 cane toads.

To investigate the relationship between V̇o_2_ and ODBA during the activity experiments, a mixed linear effects model (V̇o_2_ = ODBA + Activity Level + ODBA*Activity Level + Toad ID[random] + ODBA*Toad ID) tested for a relationship between V̇o_2_ and ODBA, and whether this relationship varied depending upon activity level and individual toad. The model was then run without the interaction terms to generate a predictive relationship between V̇o_2_ and ODBA common to all the toads included in the activity experiments.

The accuracy of the common predictive equation for the cane toads in the present study was assessed in a validation exercise, which provides quantified estimate errors and may not relate strongly to R^2^ if the modelled data do not well conform to all the relevant parametric assumptions. The same data used to derive a regression equation (i.e. V̇o_2_ and ODBA during the low and high activity experiments) can be reasonably employed to validate that equation using a jack-knife statistical technique [16], [29]. The principle is that for each individual, values of V̇o_2_ are estimated from ODBA recorded during the respirometry experiment for that animal, using a common prediction equation generated with data for that individual excluded. These V̇o_2_ estimates are then compared with the values of V̇o_2_ measured concurrently with ODBA for that animal. In the present study, for each two minute period of V̇o_2_, separately for each individual, mean V̇o_2_ and mean ODBA were placed into ODBA categories of 0–0.03 *g*, 0.03–0.06 *g*, 0.06 to 0.09 *g* and 0.09 to 0.15 *g*. Mean V̇o_2_ and mean ODBA were calculated for each ODBA category and then mean V̇o_2_ was predicted from mean ODBA, based on the common prediction equation. Actual and predicted mean V̇o_2_ were then used to calculate the mean algebraic error across individuals for each ODBA category.

Much of the individual variation in the V̇o_2_-ODBA relationship is likely to be due to differences in body mass, which is thus a factor that should be included in a common prediction equation for use with any cane toad. The data were reanalysed with body mass as a covariate in a linear effects model (V̇o_2_ = ODBA + Body mass) to produce an appropriate, common equation for any individual. The effects of body mass on the V̇o_2_-ODBA relationship were further explored by investigating the intercept and slope of the V̇o_2_-ODBA relationship for the individual toads calibrated in the present study. Firstly, single linear regressions of V̇o_2_ against ODBA during the high activity experiments were conducted for each toad individually. Secondly, the intercepts and slopes, separately, were regressed against body mass for all nine toads.

The individual regressions of V̇o_2_ against ODBA were used to estimate energy expenditure in the cane toads deployed in the field. Ambient temperature recorded on the loggers was used to Q_10_ correct estimates of V̇o_2_. The metabolic rate of ectotherms is typically strongly affected by ambient temperature, however in the species so far studied there is not an interaction between activity level and temperature. Thus the slope of the line relating metabolic rate to speed during pedestrian locomotion is independent of temperature in cockroaches *Gromphadorhina portentosa* [30], ants *Pogonomyrmex rugosus* [31], [32], lizards *Tupinambis nigropunctatus* [33], common iguana *Iguana iguana* [34], desert iguana *Dipsosaurus dorsalis* [35], and gila monsters *Heloderma suspectum* [36]. Similarly, in scorpions *Urodacus yaschenkoi*, the net cost of burrow construction (J m^−1^, calculated as the difference between burrowing and resting V̇co_2_ divided by burrowing rate) is independent of temperature [37]. In these cases, the intercept of the relationship varies with temperature according to a Q_10_ relationship [30]. Thus, the slope of the relationship between V̇o_2_ and ODBA in cane toads is assumed to be independent of temperature while the intercept is assumed to vary with temperature with a Q_10_ of 2.49 [38], which is slightly higher than the average Q_10_ for amphibians (2.21; White et al. 2006a).

**Figure 1:**
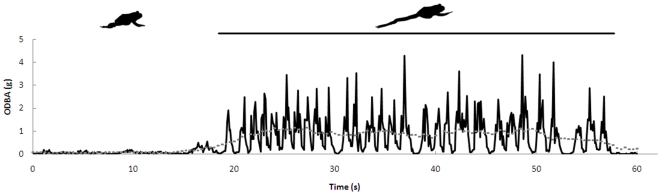
Values of ODBA measured in a free-ranging cane toad. The ODBA trace (black) while the toad is hopping is clearly different to the trace while it is fairly inactive. The grey stippled line represents mean ODBA per five second period, which was used in a computer program to determine five second periods when the toad was hopping (mean ODBA >0.3 *g*; denoted by the black bar) or not hopping.

Mean V̇o_2_ over the entirety of the recording period while the animal was in the field was calculated, along with maximum V̇o_2_ over a 5 minute period. Through scrutiny of the ODBA traces from short logger deployments outside the laboratory while toads were allowed to be non-active, then encouraged to move a little and then to hop (unpublished data but see Figure 1), it was possible to determine threshold values of ODBA defining which of these three behavioural types a cane toad was exhibiting at any given time. Because spikes in acceleration readings can occur for a number of reasons other than due to these activities directly, such as falling from a small rock or the logger hitting a low hanging branch, ODBA must be averaged over a biologically meaningful period of time before being interpreted. In the present study, mean ODBA was calculated for the field data for each consecutive five second period. Per five seconds, behaviour was defined as non-activity when mean ODBA was less than 0.075 *g*, low activity behaviour (e.g. movements changing body orientation, slowly burrowing into the substrate) when mean ODBA was between 0.075 and 0.3 *g*, and hopping when mean ODBA was greater than 0.3 *g*. Using a custom made program written in Matlab (v 6.5, The MathWorks), including the threshold ODBA values described above, the behavioural time budgets of the toads while in the field were calculated. Furthermore, the mean rate of energy expenditure was calculated for the behaviours classed as non-activity and low-activity, for each toad individually, by applying the individual-specific calibrations to the mean ODBA values for each behaviour. Rate of oxygen consumption during hopping was not calculated since anaerobic metabolism is known to contribute significantly in supporting unsustainable, short term activity [39].[Fig pone-0010170-g002]

**Figure 2 pone-0010170-g002:**
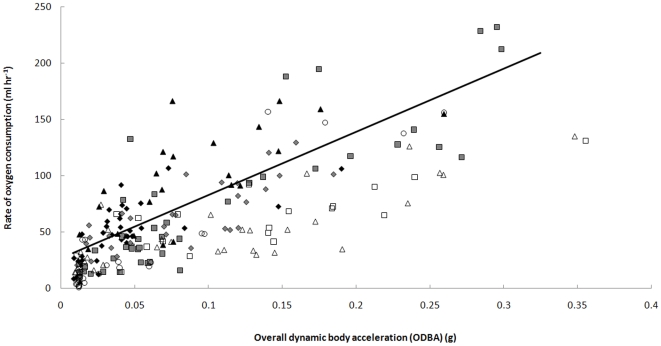
Rate of oxygen consumption against ODBA for nine cane toads during a range of activity levels. The common slope shown, derived from a linear mixed effects model, is described by y = 9.344 * x + 0.451.

## Results

### Resting metabolic rate in the laboratory

A linear regression of resting V̇o_2_ in the laboratory against body mass was significant (P<0.001):
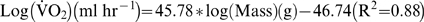
(1)Mean resting V̇o_2_ in the laboratory for the eight toads from which field data were obtained was 8.28 ml O_2_ hr^−1^ (range: 4.44 to 12.9) at 25°C.

### Validation exercise

A mixed effects linear model (V̇o_2_ = ODBA + Activity Level + ODBA*Activity Level + Toad ID[random] + ODBA*Toad ID; R^2^ = 0.81) indicated a significant relationship between V̇o_2_ and ODBA (P<0.001) but no interaction between ODBA and activity level (P = 0.574). Thus the relationship between V̇o_2_ and ODBA did not vary during low or high activity and the interaction term was removed. There was an interaction between ODBA and Toad ID (P<0.001) indicating that the slopes of the V̇o_2_-ODBA relationships varied between individual animals. The same model with the interactions removed was used as the common prediction equation for the validation exercise, i.e. for all the instrumented toads in the present study:
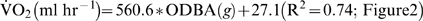
(2)The validation exercise returned a mean algebraic error of −15.2% (range: −82.2 to 29.0%) for mean ODBA between 0 and 0.03 *g*, 4.2% (range: −44.7 to 25.6%) for mean ODBA between 0.03 and 0.06 *g*, 1.1% (range: −43.2 to 23.1%) for mean ODBA between 0.06 and 0.09 *g*, and −0.9% (range: −48.5 to 29.7%) for mean ODBA between 0.09 and 0.15 *g*.

Based on the present data, the equation for predicting V̇o_2_ of any cane toad from ODBA is:
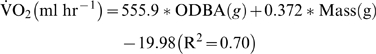
(3)


### Scaling of calibrations

To test for associations between the relationships of V̇o_2_ against ODBA and toad size, the slopes and intercepts of the high activity relationship for each individual toad were regressed against body mass (g). Intercept was significantly, positively related to body mass (intercept = 0.0060×body mass – 0.1220; R^2^ = 0.71) and slope was also significantly, positively related to body mass (slope = 0.0403×body mass + 1.7794; R^2^ = 0.41).

### Quantifying behaviour and metabolic rate during field deployments

The behavioural and energetic time budgets of eight toads while in the field are shown in Tables 1 and 2. Table 1 provides the deployment details along with general mean ODBA and energetics values. Table 2 details the behavioural time budgets of each toad (the duration, and proportion of time, spent hopping, exhibiting low level activity, or non-active), along with estimated rate of energy expenditure during each of these behaviours apart from hopping. The modal behaviour was non-activity, and hopping typically occurred for less than 5% of the time. Mean estimated V̇o_2_ over the recording period was 5.5 times resting V̇o_2_ measured in the laboratory (Q_10_ correcting to the same temperature). Maximum estimated V̇o_2_ over a five minute period was nearly 9 times measured resting V̇o_2_ in the laboratory (Q_10_ correcting to the same temperature).

**Table 1:** Field deployments of accelerometry and temperature loggers, calculated ODBA values and estimated rates of energy expenditure (rate of oxygen consumption; V̇o_2_).

Toad ID	Body mass at time of deployment (g)	Location	Duration logger deployed on animal (hh∶mm)	Mean temperature over recording time (°C)	Mean ODBA over recording time (*g*)	Mean estimated V̇o_2_ over deployment time (ml hr^−1^, Q_10_ corrected)**	Maximum ODBA over five min (*g*)	Maximum estimated V̇o_2_ over five min (ml hr^−1^, Q_10_ corrected)
a	98	Cricket ground	3∶17	16.5	0.054	22.6	0.114	33.0
c	175	University garden	1∶10	19.3	0.0439	42.6	0.058	47.4
d	112	Cricket ground	4∶15	16.1	0.0518	22.6	0.115	35.5
f	135	University garden	5∶10	18.5	0.0249	11.8	0.063	29.1
f	133	University garden	2∶48	14.0	0.0487	15.1	0.174	52.8
g	97	Football ground	2∶47	13.4	0.0302	8.8	0.080	14.0
h	133	University garden	1∶15	19.7	0.0301	31.8	0.060	38.4
i	204	University garden	0∶19	19.3	0.0232	29.9	0.028	31.1
Mean ± SEM	136±13		2∶37±0∶34	17.1±0.9	0.0384±0.0044	23.1±4.0	0.086±0.016	35.2±4.2

*From the time of deployment until recovery of the logger, unless the logger had already stopped.

**Including periods of hopping, which may include significant portions of anaerobic metabolism (see text).

**Table 2:** Behavioural time budgets and associated estimated rates of energy expenditure (rate of oxygen consumption; V̇o_2_) obtained from accelerometer field deployments.

Toad ID	Location	Proportion of recording time spent resting (%)	Proportion of recording time spent undertaking low activity behaviour (%)	Proportion of recording time spent hopping (%)	Mean estimated V̇o_2_ during resting (ml min^−1^, Q_10_ corrected)	Mean estimated V̇o_2_ during low level activities (ml min^−1^, Q_10_ corrected)	Multiple of resting V̇o_2_
a	Cricket ground	74.8	22.1	3.1	16.7	40.8	2.4
c	University garden	81.3	17.3	1.4	37.2	70.8	1.9
d	Cricket ground	77.3	19.2	3.5	15.3	48.2	3.2
f	University garden	91.6	8.2	0.1	8.8	62.1	7.1
f	Football pitch	83.1	10.6	6.3	4.2	59.5	14.2
g	Football pitch	88.9	9.8	1.3	7.0	24.5	3.5
h	University garden	79.1	19.9	1.0	30.5	54.8	1.8
i	University garden	96.0	4.0	0.0	29.9	52.9	1.8
Mean ± SEM		84.0	13.87±2.3	2.10±0.7	18.7±4.4	51.7±5.0 (2.8)	2.8±1.5

## Discussion

Much research has gone into understanding the effects of introduced organisms on native environments and species [3]. Often valuable to this endeavour is valid measuring of the energy expenditure of those alien animals to understand their energy requirements [e.g. 40], since habitats with greater abundance of suitable resources are likely to result in their higher reproductive success and recruitment, and lower mortality. However, while a number of anuran species have been introduced to foreign locations [e.g. 41], [42], with the cane toad perhaps the most infamous example, few data exist on the field metabolic rates of this taxon. Cane toads move on average around 4% of the time and 18 m per hour, though this varies considerably depending upon a range of factors [26], [43]. Further, in Fowler's toad *B. woodhousei fowleri*, maximum V̇o_2_ during treadmill exercise was nine times pre-exercise V̇o_2_ [39], thus during periods of free-ranging locomotion, it is possible that metabolic rate in *Bufo* species greatly increases. Thus presently, field metabolic rates of toads and frogs are very unclear and can only be estimated [26]. However, the current study indicates that the accelerometry technique represents a tractable method for obtaining valid estimates of metabolic rate in anurans, in particular as progressively smaller loggers are developed.

As has been found in previous studies calibrating metabolic rate with ODBA, the relationships for cane toads were positive and strong. Further, the mean algebraic errors of the validation exercise were small indicating that the accuracy of ODBA for measuring V̇o_2_ in cane toads is comparable to previous studies using ODBA or heart rate for measuring mean V̇o_2_ for a group of individuals of other species [15], [16 and tables within]. The validity of the calibrations is further supported by the significant relationships between the intercept and slope of each individual calibration, and body mass. As stated in Halsey et al. (2009c), such relationships would be predicted based on expectations of metabolic rate scaling [44], [45].

### Accelerometry for measuring anuran field energy expenditure

Mean metabolic rate of the cane toads during the field deployments (Table 1) was around 5 times higher than their resting metabolic rate recorded in the laboratory after Q_10_ correcting to the same temperature. This is considerably greater than the resting metabolic rate multiple of 2.5 assumed by Kearney et al. (2008) to estimate field metabolic rate, from which they predicted feeding rates for inclusion in a model of future cane toad distributions. In the present study, deployments were undertaken at night and during rainy or at least ground-wet conditions; environmental conditions that encourage movement from cane toads [46] (during the four nights of deployments a severe weather warning concerning heavy rains was issued by the Australian Government Bureau of Meteorology). Furthermore, cane toads may tend to exhibit more locomotion behaviour in the period directly after release. On the other hand, rainy days also tend to have a lower ambient temperature (Kearney et al. 2008; mean temperature in the present study during field deployments was 17.1°C), typically reducing anuran activity levels. Thus the present data, while too short term to be assumed as indicative of typical levels of energy expenditure in wild cane toads, at least indicate that previously used estimates of metabolic rate for this species could be inaccurate. It may be that cane toad energy expenditure in the wild can be considerably higher than might be assumed based on, for example, the multiple of resting metabolic rate that is typically the case in mammals (average 2.65) [47]. Indeed, greatest mean metabolic rate in the field over five minutes was 9 times resting metabolic rate in the laboratory (Q_10_ correcting to the same temperature). This multiple matches that reported for Fowler's toad in the laboratory, which is further evidence for the validity of the estimations obtained in the present study.

During the field deployments the toads were post-absorptive since they had not eaten for a number of weeks and SDA continues for between around five and ten days in *Bufo* species after a large meal [38]. Accelerometry is not sensitive to increases in energy expenditure due to SDA (cf. Green et al. 2009). Thus when using the technique to gain valid estimates of field energetics in species that exhibit significant increases in post-prandial metabolism, estimates of energy expenditure due to SDA must be added to the values of energy expenditure derived from the logger data. From calculations of resting V̇o_2_ obtained in the laboratory (equation 1), a 120 g cane toad at 25°C in the field will consume a minimum of 6.4 ml O_2_ hr^−1^, which represents about 3200 J day^−1^. Assuming a diet of crickets, which have an energy density of around 6.3 kJ g^−1^ wet mass [48] and an assimilation efficiency of 85% (Kearney et al. 2008), the cane toad would need to eat about three grams of food every 5 days to provide sufficient energy to compensate for that lost in a resting, postprandial state. The relationship between SDA and energy ingested is close to isometric in cane toads (Secor and Faulkner 2002) thus whether a cane toad eats small meals frequently or large meals infrequently does not affect total SDA, while SDA increases in unitary proportion with estimates of metabolic rate. For example, over 50 days, estimated energy expenditure based on resting data would be about 160 kJ and energy expenditure due to SDA would be about 61 kJ (based on ingestion of a total of 30 g of cricket and extrapolating from the nearest values available in Table 1 of Secor and Faulkner 2002, and assuming digestion was complete at 50 days). This represents an increase in metabolic rate over the minimum that could be estimated from the accelerometry and temperature data alone of around 34%. Thus for long term field deployments, energy expenditure estimated from the logger data could be augmented by an estimated amount as calculated above. Different values may need to be used when calculating SDA depending upon ambient temperature and food type though the former factor does not seem to have a great deal of effect [38], [49].

### Accelerometry for obtaining behavioural-energy time budgets

Quantified behavioural and energetic information combined are invaluable for understanding an animal's ecology and, in turn, the limitations of invasive species in terms of the habitats they can tolerate and the likely spread of populations. Accelerometry is a developing technique for obtaining both types of data [13], [15], [18], [19]. Figure 1 provides an example for cane toads. There is clearly a distinct trace in ODBA over time during periods when a toad is hopping and thus ODBA traces can be used to describe these periods. In the present example, periods of low level activity (slow movements) and non-activity were also described. Furthermore, such analysis allows the total duration of each type of behaviour to be calculated along with mean ODBA during the entirety that each behaviour is exhibited, the latter then being used to estimate behaviour-specific energy expenditures.

During the field deployments in the present study, the toads spent on average around 16% of the time being active, during which they were hopping for about 13% of the time. This is a similar proportion of overall time spent hopping as was reported from behavioural observations by Kearney et al. (2008), suggesting that the behavioural effects of the relatively large loggers on the instrumented animals were limited. Rate of oxygen consumption during low-level activity were about 3-fold that during non-activity suggesting that changes in behavioural time budgets can have a marked effect on energy expenditure. These data represent the first time that accelerometers have been used not only to quantify the behaviour of free-ranging animals but also to assign to them estimated rates of total energy expenditure.

### Methodological Improvements

In the current study the loggers were large relative to the size of the toads. Smaller animals can carry relatively larger masses than can larger animals [50], making invalid the general use of the ‘5% rule’ [51] across species, and the instrumented cane toads in the present study did not hop for a clearly different proportion of time compared to that previously reported. Nonetheless the accelerometers used probably significantly increased the cost of activity [52], resulting in higher rates of energy expenditure in the field than would be otherwise exhibited. Fortunately, accelerometry-based data loggers are progressively being further miniaturised and in the foreseeable future should be small enough to deploy on cane toads without affecting energy expenditure during movement. Already in 2010, for example, Cefas Technology Limited are producing triaxial accelerometers weighing 18 g including battery, and loggers of similar dimensions can presently record for a week [53]. It should be noted that Equation 3 may tend to overestimate energy expenditure from data recorded using a smaller logger.

Ideally, temperature measurements would record subcutaneously rather than externally since there can sometimes be some variation between the two in toads [25]. An enhanced method of attachment, and perhaps also a flatter logger, needs to be developed if longer field deployments are desired, such that the logger remains in place indefinitely while still not damaging or irritating the animal. Alternatively, appropriately miniaturised loggers could be surgically implanted, as is routine for the heart rate technique [11].

The metabolic cost of SDA over many days can be reasonably estimated based on the metabolic rate predicted from accelerometry and temperature indicating food requirements coupled with previous papers measuring SDA in the laboratory. That estimate of post prandial metabolic rate can then be added to the accelerometry-based prediction of metabolic rate to provide a more accurate estimate of field energy expenditure. However, over shorter deployments (e.g. a few days) the magnitude of SDA cannot be calculated. Information would be required on the size of meals recently ingested by the subject toad, and also when these ingestions took place since the magnitude of SDA after a meal varies considerably over subsequent days [38]. Thus the methodology of estimating field energetics presented in the current study is only suitable for long-term deployments, or short-term deployments where subject animals are known to be post-absorptive. However, post prandial metabolic rate is not a factor when specifically measuring the biomechanical costs of active animals, and ODBA has been used effectively to this end with diving cormorants [54]. In a similar fashion, ODBA could be particularly useful for assessing the costs of movement in cane toads of differing morphology, e.g. those at invasion fronts tend to have longer legs than those in established populations [1].

### Conclusions

A quantified understanding of the ecology of a species is required for effective conservation management, either to protect that species or to limit the damage it causes when introduced to new locations. The accelerometry technique provides key information on two fundamental aspects of animal ecology; behavioural time budgets and, along with estimates of SDA and temperature effects, associated energy expenditure. The present study demonstrates its suitability for use with medium and large ectotherms, through application of the method to a taxonomic group for which previous estimates of field energetics and behavioural time budgets have been obtained only through modelling and observation, or vocal recordings [e.g. 55], respectively. Clearly, measuring energy expenditure and behaviour in small ectotherms is difficult due to present logger sizes and temperature effects on metabolism, however technology is rapidly addressing the former while the latter can be accounted for. The stage is now set for detailed studies of the combined behavioural and metabolic budgeting of species and individuals in the field, using the accelerometry technique as the linchpin.
